# Trends in prevalence and predictors of anemia among school-aged children in Tanzania: analysis of 2019–2023 cross-sectional surveys using a multilevel logistic regression model

**DOI:** 10.1186/s12889-026-27799-y

**Published:** 2026-05-27

**Authors:** Frank Chacky, Susan F. Rumisha, Mbaraka John Remiji, Sijenunu Aaron, Nyabasi Makori, Fabrizio Molteni, Patrick GT Walker, Wiggins Aaron Kyatikila, Felista Mwingira, Bwire Wilson, Pendael Machafuko, Agnes A. Mpinga, Grace Moshi, Theresia Jumbe, Nyamizi Bundala, Ester Kawishe, Joseph T. Hicks, Geofrey Mchau, Samson Kiware, Germana Leyna, Witness Mchwampaka, Witness Saitot, Oliva Kimaro, Julieth Silao, Samafilan Ainan, Geofrey Makenga, Humphrey Mkali, Achyut KC, Prosper Chaki, Billy Ngasala, Samwel L. Nhiga, Robert W. Snow, Jean-Pierre Van Geertruyden, Bruno P. Mmbando

**Affiliations:** 1 Ministry of Health, Dodoma, Tanzania; 2 National Malaria Control Programme, Dodoma, Tanzania; 3 Global Health Institute, University of Antwerp, Antwerp, Belgium; 4 Ifakara Health Institute, Dar es Salaam, Tanzania; 5 National Institute for Medical Research, Dar es Salaam, Tanzania; 6 The Kids Research Institute, Perth, Australia; 7 Tanzania Food and Nutrition Centre, Dar es salaam, Tanzania; 8 Swiss Tropical and Public Health Institute, Dar-es-salaam, Tanzania; 9 Swiss Tropical and Public Health Institute, Basel, Switzerland; 10 Imperial College London, London, UK; 11 Department of Statistics, University of Dar es salaam, Dar es Salaam, Tanzania; 12 Biological Sciences Department, Dar es Salaam University College of Education, Dar es Salaam, Tanzania; 13 National Institute of Medical Research, Tanga, Amani Tanzania; 14 Centre of Excellence in Health Monitoring and Evaluation, Mzumbe University, Morogoro, Tanzania; 15 Sokoine University of Agriculture, Morogoro, Tanzania; 16 Morogoro Municipal Council, Morogoro, Tanzania; 17 National Bureau of Statistics, Dodoma, Tanzania; 18 Department of Paediatrics and Child Health, School of Medicine, Muhimbili University of Health and Allied Sciences, Dar es Salaam, Tanzania; 19 National Institute for Medical Research, Tanga Center, Tanga, Tanzania; 20 Population Service International, Dar es Salaam, Tanzania; 21 Department of Microbiology, Tumor and Cell Biology, Karolinska Institute, Stockholm, Sweden; 22 Muhimbili University of Health and Allied Sciences, Dar es Salaam, Tanzania; 23 Ubuntu Health, Atlanta, Georgia USA; 24 KEMRI-Wellcome Trust Research Programme, Nairobi, Kenya; 25 Centre for Tropical Medicine & Global Health, Nuffield Department of Medicine, University of Oxford, Oxford, UK

**Keywords:** Anemia, School age children, Tanzania malaria survey, Anemia prevalence, Surveillance, Anemia burden

## Abstract

**Background:**

Anemia constitutes a major global public health challenge among school-aged children with documented impacts on psychomotor development, academic achievement, susceptibility to infections, and long-term economic productivity. This study presents nationally representative findings on the prevalence and determinants of anemia among school children aged 5–16 years in mainland Tanzania.

**Methods:**

Data were collected as part of the cross-sectional Tanzania School Malaria Parasitological Survey (SMPS) in 2019, 2021, and 2023. Hemoglobin concentration was assessed using the HemoCue Hb 201 + analyzer, and anemia status was classified according to the 2024 WHO thresholds. Candidate predictors associated with anemia (*p* < 0.2 in univariable analyses) were included in a multivariable multilevel logistic regression model to assess the contribution of predictor variables to anemia prevalence and to calculate adjusted odds ratios (AOR) and 95% confidence intervals (CI).

**Results:**

The study surveyed 47,366 school-aged children from 810 schools across three survey rounds. Generally, anemia prevalence was 45.5% (mild: 18.5%, moderate: 25.4%, severe: 1.5%). On multivariable regression of anemia risk, a statistically significant interaction was observed between age and sex. While odds of anemia were similar between sexes in lower age groups (5–11 and 12–14 years), male children aged 15–16 years old had almost three times the odds of anemia compared to female children in the same age group (AOR 2.96 [95%CI 2.18–4.02]). Malaria infection significantly increased anemia odds (AOR 1.88 [95%CI 1.75–2.01]). Regional disparities were noted, with the Lake [AOR 1.77 (95%CI 1.38–2.26)] and Western [AOR 1.78 (95%CI 1.34–2.37)] zones having highest odds of anemia compared to the Central zone. Over time, anemia risk decreased, with a notable reduction in 2023 (AOR 0.75 [95%CI 0.70–0.80]) compared to reference year (2019).

**Conclusions:**

Anemia in school-aged children remains a severe public health problem in Tanzania, according to WHO classifications. Risk factors include older age, male sex, malaria infection, and living in moderate and high malaria transmission risk areas and zones like the Lake, West, and Southern. These findings highlight the need for collaborative efforts by stakeholders to address anemia not only in Tanzania but also in regions with similar challenges.

**Supplementary Information:**

The online version contains supplementary material available at 10.1186/s12889-026-27799-y.

## Introduction

Globally, anemia is a major public health concern, affecting younger children aged 0–5 years (40%), pregnant women (30%), and women of reproductive age (15–49 years) (37%); as well as people in lower-middle income countries, especially those who resides in rural areas and with low education levels [1, 2]. A global trend analysis of anemia burden by severity and cause reported high prevalence (41·4%) among under-five years, with higher rates observed in female (31·2%) than male (17.5%) children [3, 4]. Globally, estimated cases of anemia increased by 6.7% in 2019 (1.8 billion) to 1.92 billion in 2021 [3, 4].

Anemia is a global public health problem among school-aged children (SAC) and adolescents in countries with the lowest socio-demographic Index (SDI), especially in South Asia and sub-Saharan Africa (SSA) [4, 5]. Nearly, a quarter of school children surveyed in Ethiopia were reported to be anemic [6] whereas a study conducted in Kenya reported increased anemia prevalence with increasing age [7]. SAC may be particularly vulnerable to anemia due to physiological changes and increased nutritional requirements during this critical stage of growth and development [8]. Anemia among school children has been reported to impair psychomotor development, mental growth, reduced school performance, increased infection risks, and loss of productivity at a later age in life [6, 9].

SAC may be identified as anemic when hemoglobin (Hb) concentration level falls < 11.5 g/dL for children aged 5–11 years (male & female), < 12.0 g/dL for female and male children aged 12–14 years, and < 12.0 g/dL for female children aged 15–16 years, while males aged 15–16 years are considered anemic if their Hb is < 13.0 g/dL [10]. Anemia may occur as a manifestation of the underlying cause such as nutritional deficiencies (e.g., iron deficiency), chronic diseases, genetic disorders affecting red blood cell production, or blood loss due to injury or other medical conditions including lung and liver diseases, drug induced, hemolysis, and parasitic diseases, among others [2, 3, 11].

While iron deficiency accounts for 50% of anemia cases [3], malaria and other parasitic diseases have been reported as the major contributor of anemia, especially in areas where their infections are endemic [12, 13]. In Tanzania, most studies on anemia among SAC have been geographically or thematically limited, offering insights into specific populations while lacking generalizability across the country. For example, a study in Zanzibar reported an anemia prevalence of 53.3% among adolescents aged 10–17 years, highlighting regional disparities and the need for targeted interventions [14]. Another study underscored the potential for school-based anemia prevention strategies in Tanzania, particularly through nutrition education and iron supplementation [15]. Mrimi et al. [16] found a 43.4% prevalence of anemia among SAC in rural areas, while a multi-country analysis covering Ethiopia, Sudan, and Tanzania identified regional variations in anemia burden, with notable risk differentials among Tanzanian adolescents [17].

These studies also identified key determinants of anemia, including low household wealth [14, 17], iron deficiency and undernutrition [16], and co-infections with malaria and soil-transmitted helminths [16]. Additionally, gender-related disparities were commonly observed, with girls at higher risk of anemia [14, 17]. Other context-specific factors, such as poor sanitation and regional disparities, were highlighted in Zanzibar [14], whereas parasitic co-infections and rural-urban differentials were noted in mainland Tanzania [16].

Despite these findings, previous research in Tanzania has limitations. Many studies focused on adolescents aged 10–17 years [14, 17] neglecting younger SAC aged 5–9 years. Furthermore, most analyses were conducted in specific regions or involved multi-country comparisons, with few employing multilevel models to account for nested data structures, thus limiting their policy relevance for nationwide intervention planning.

There remains a critical gap in nationally representative data on anemia among SAC in Tanzania, particularly for informing interventions and policies at regional and council levels. This paper addresses that gap by analyzing nationwide primary collected data from 184 councils across all 26 regions through Tanzania School Malaria Parasitological Survey (SMPS). SMPS is a surveillance system aimed at monitoring malaria prevalence and related health outcomes among SAC (5–16 years). The SMPS goals include informing malaria control strategies, evaluating intervention impact, and incorporating a broad range of predictors, including parasitic infections, intervention coverage, and household-level socioeconomic characteristics.

By applying multilevel modeling to account for contextual variability, this study provides novel insights into the prevalence, severity, and risk factors of anemia among SAC across Tanzania’s diverse geographic and epidemiological settings. Despite the public health importance of anemia in SAC driven by high nutritional demands and exposure to parasitic infections such as malaria; nationally representative data remain limited to inform targeted interventions and policy decisions. A more comprehensive understanding of the trends and determinants of anemia in SAC is essential to guide evidence-based prevention and management strategies. Therefore, this study aimed to assess the prevalence and severity of anemia and to identify associated risk factors among children aged 5–16 years using data from large–scale, nationwide school-based SMPSs.

## Methods

### Study area and survey design

Mainland Tanzania is characterized by diverse and complex topographical features extending from a narrow coastal belt of the Indian Ocean with an extensive plateau and elevation ranging from 1,000 m to 2,000 m above sea level (m asl). About 75% of the Tanzanian population lives in areas above 500 m asl [18]. According to the 2022 Tanzania National Population and Housing Census (NPHC); the country had a population of 59,851,347 of which 31.6% were children aged 5–16 years old [19].

Mainland Tanzania is divided into 26 regions serving the first level of administrative division and 184 councils serving as the second-level administrative units responsible for governance, public service delivery, and development planning at the local level. Councils (subdivisions of the regions) are categorized as City Councils (urban centers), Municipal Councils (growing urban areas), Town Councils (smaller urban centers), and District Councils covering rural and semi-urban areas. Wards are subdivisions of councils and serve as third-level administrative units, acting as links between local government authorities and communities.

The SMPS is a biennial cross-sectional school survey targeting children aged 5–16 years enrolled in public primary schools across all 184 councils of mainland Tanzania [20]. To date, five consecutive surveys have been conducted in 2014-15, 2017, 2019, 2021, and 2023 to monitor malaria prevalence and intervention coverage. Since the 2019 survey round, SMPS has introduced a collection of nutrition indices such as anemia, Body Mass Index (BMI) for stunting, dietary diversity score, food security, and associated risk factors.

To be included in the survey, a child must be between the ages 5–16 years, enrolled in public primary schools, present on the survey day, have parent permission through an opt-out approach, and provide a verbal assent on the day. SMPS is a school-based surveillance system in which the primary unit of analysis is the school rather than individual pupils; therefore, unique identifiers are not routinely used to track individuals across survey rounds.

### Sample size determination and sampling

The survey applied probability proportional to size (PPS) sampling, whereby schools and pupils were selected from each council in proportion to the council population. This resulted in an approximately self-weighting design, with larger councils contributing proportionally more observations, thereby reflecting the national population distribution. The study was designed to achieve council-level representativeness, with schools systematically sampled from wards across all councils in mainland Tanzania. As the survey was restricted to public primary schools with relatively comparable enrollment, council population was used as a proxy for the school-going population; therefore, explicit post-survey weighting was not applied. Multilevel models with random effects at school and ward levels accounted for the hierarchical structure of the data.

Sample size was estimated at council level based on malaria prevalence estimates from the preceded surveys and population size. The estimated council sample sizes were aggregated to obtain a national sample of 63,806; 64,959 and 65,540 for 2019, 2021, and 2023 respectively. Of these, 33% in 2019 and 2021 and 10% in 2023 children were systematically selected for Hb concentration measurements. Each school’s Hb level measurement sample size was divided equally among the seven classes/grades and sex. A list of children selected for malaria testing per class served as a sampling frame from which individual students were randomly selected for Hb level measurement.

### Consenting procedures

Permissions for the study were obtained from the Prime Minister’s Office Regional Administration and Local Government (PMO-RALG) before approaching councils and selected schools. District Education Officers (DEOs), Ward Education Officers, and head teachers at each selected school were briefed on the survey and provided with an information sheet outlining the survey procedures. Head teachers were requested to organize meetings with school committee members, teachers, and field teams to present the study’s objectives, explain the organization and implementation plan, address any questions, and outline the potential benefits, possible risks, and the voluntary nature of participation including the right to withdraw at any point without consequence. School committees were asked to provide formal permission for their schools to participate. Upon agreement, approval was documented through signed meeting minutes.

Individual written parental consent was not sought since the survey was conducted under the auspices of the Tanzania Ministry of Health which has the legal mandate to conduct routine malaria surveillance. As such consent for the survey was based on a passive, opt-out method of parental permission. This approach is an ethical and practical way of informing participants in low-risk studies and interventions [21] and has been used in several school-based studies, including studies in the United Kingdom, United States, India and Kenya [22–26].

Once a child was selected to participate in the survey, schoolteachers sent a memo to parents/guardians requesting their consent through a passive, opt-out approach. The memo clearly outlined that parents/guardians had the option to decline their child’s participation by explicitly signing and returning the memo to indicate non-consent. The memo information on the purpose of the survey, the procedures such as finger-prick blood testing, interviews, and potential referrals and/or provision of antimalarial medicines for sick children along with the potential risks and benefits of participation. The memo assured parents of the confidentiality of all collected data and emphasized that declining participation would have no negative consequences. Additionally, verbal assent was obtained from each selected child before their participation in the survey. If a parent or guardian chose not to allow their children to participate in the survey, the child’s name was removed from the school rolls. On the day of the survey, the survey team leader informed all children at the school about the sampling and survey procedures, making it clear to their participation was entirely voluntary and that they may opt out of the testing at any time without consequence. Any child with an acute illness was excluded from the study.

### Training of the data collectors and data collection

Council-level data collectors composed of malaria focal persons, school health coordinators, and practicing laboratory technicians were identified by each council. A structured and adaptive training curriculum was implemented to align teams with evolving survey objectives and ensure high-quality, standardized data collection. Training modules covered survey design, sampling methodology, malaria testing, hemoglobin concentration estimation, dried blood spot (DBS) collection, anthropometric assessments, interviewing techniques, informed consent, data quality assurance, ethical protocols, financial documentation, and procedures for data transport and storage. Practical sessions, including field pre-testing and role-playing, were integrated to reinforce understanding and consistency across teams. This comprehensive training model played a crucial role in operational efficiency and maintaining data integrity across all survey rounds.

Supervisory roles were fulfilled by designated Regional Malaria Focal Persons and Laboratory Technologists, supported by the national team from NMCP and partner institutions, who were responsible for field oversight, protocol compliance, and verification of submitted data.

Data were collected during the following periods: from August to September 2019 from 646 schools, from October to November 2021 from 687 schools, and from August to October 2023 from 651 schools. The data collection tools used in this study were part of the national programmatic surveillance under SMPS [20, 27]. The children were interviewed for demographic and household characteristics including age, sex, household size, history of fever in the past 14 days, and use of mosquito nets the night preceding the survey. To ensure the reliability of self-reported data from younger children (aged 5–9 years), interviewers supported by two selected teachers from each surveyed school applied age-appropriate questioning techniques. All field teams received specific training on effective communication with young children. During interviews with early primary school pupils, teachers were present to assist in rephrasing or clarifying questions using locally familiar language and concepts. The questionnaire focused on concrete and recent experiences, such as whether the child slept under a mosquito net the previous night or had experienced fever in the past two weeks, using locally understood terms for fever and illness to enhance comprehension and accuracy.

The children selected for Hb level measurements were finger pricked to collect a drop of peripheral blood in a microcuvette to measure the Hb concentration using a portable battery-operated HemoCue Hb 201 + analyzer with a precision of +/- 0.3 g/dL as recommended by WHO for field surveys [28]. The same HemoCue Hb 201 + analyzer was consistently used to measure hemoglobin concentration levels among SAC across all three SMPSs. Height was determined using a Shorr Board and recorded to the nearest 0.1 cm whereas weight was measured using a SECA scale; an electronic weighing scale which was calibrated daily using a 2 kg iron bar and was recorded in kilograms to the nearest 0.1 kg. Body mass index (BMI) was defined as a quotient of the body weight in kilograms per square meter of height (kg/m^2^). Malaria infection was assessed using a CareStart Malaria Pf/PAN (HRP2/pLDH) Ag rapid diagnostic test (RDT).

Children with malaria positive test results were treated with artemether–lumefantrine (ALu) according to the National Malaria Diagnosis and Treatment Guidelines [29]. When necessary, children with severe illnesses including anemia were referred to the nearest health facility for further management.

## Variable definitions

### Dependent variable

In this study, the outcome variable was the anemia status of SAC (5–16 years).

For regression analyses, anemia status was modelled as a binary variable: anemic [1] vs. non-anemic (0), using WHO 2024 age- and sex-specific haemoglobin thresholds [10]. Children were classified as anemic if their altitude-adjusted haemoglobin fell below the relevant cutoff: <11.5 g/dL (ages 5–11), < 12.0 g/dL (ages 12–14 and girls aged 15–16), and < 13.0 g/dL (boys aged 15–16).

Anemia severity was further categorised at the individual level by calculating the difference between each child’s haemoglobin value and their threshold. Severity levels were defined as: mild (0 to < 2 g/dL below threshold), moderate (2 to < 3 g/dL below), and severe (≥ 3 g/dL below threshold). These classifications were used to define three binary outcomes for mapping and sub-group analysis: any anemia (mild or worse), moderate-or-severe anemia, and severe anemia alone. Although the data for this study were collected prior to 2024, we applied the most recent WHO anemia cut-offs [10] to classify anemia status in order to maintain consistency with current global standards and enhance comparability with future research; this approach is supported by WHO recommendations for the retrospective application of updated diagnostic thresholds [10].

School children who had Hb concentration levels of less than 8.0 g/dL were referred to the nearby health facilities for further investigation and management. A child with a positive malaria test result was treated with artemether–lumefantrine (ALu), as recommended in the National Malaria Diagnosis and Treatment Guidelines [29] and when deemed necessary was referred to the nearest health facility.

## Independent variables

### Child-level variables

School children were categorized into three age groups: young children (5–11 years), early adolescents (12–14 years), and late adolescents (15–16 years). Sex was categorized as male and female. The use of mosquito nets the night before the survey was coded as yes, and no. Height-for-age Z-scores were used to classify children as stunted (Z-score ≤ -2) or non-stunted (Z-score > -2). Regarding nutritional status, children were classified using the WHO AnthroPlus^®^ 1.0.4 software [30] based on Weight-for-age Z-scores, which categorized children as Normal (Z-score − 1 SD to + 1 SD), Underweight (Z-score < -2 SD), and Thin using Body Mass Index (BMI)-for-Age Z-scores (BAZ) (Z-score < -2 SD). Malaria infection was categorized as a positive or negative RDT result.

### School/household-level variables

School altitude, measured in meters above sea level (m asl), was categorized into four groups: lowland (< 750 m asl), midland (750–1250 m asl), highland (1250–1750 m asl), and mountainous (> 1750 m asl). Household size was classified into two categories: small households (four or fewer members) and large households (more than four members) [19]. Based on the 2022 national census data, at the Ward level, schools were classified by location into rural, semi-rural, or urban areas, an indicator referred to as residence [19].

### Malaria exposure variables

Malaria infection status was determined at the individual level using rapid diagnostic tests (RDTs), with results classified as either positive or negative. This variable was included as a proximate indicator of current or recent malaria infection.

To complement this, council-level malaria endemicity strata were included as a contextual covariate. These were based on the standard stratification used by the Tanzania National Malaria Control Programme and classified each council into one of four epidemiological categories: very low, low, moderate, or high to transmission risks [31]. Endemicity strata were incorporated as a marker of longer-term ecological exposure risk, to capture background variation in cumulative malaria exposure and acquired immunity. This allowed us to reflect both immediate and contextual aspects of malaria-related anemia risk, which may act through distinct biological pathways.

### Anemia severity and public health significance

Anemia severity at the individual level was defined according to WHO thresholds: mild (11.0–11.4 g/dL), moderate (8.0–10.9 g/dL), and severe (< 8.0 g/dL).

To describe the population-level burden of anemia, we applied WHO thresholds for public health significance based on prevalence: <5% (no public health problem), 5.0–19.9% (mild), 20.0–39.9% (moderate), and ≥ 40% (severe) [32].

### Statistical analysis and model evaluation

We employed a multilevel mixed-effects logistic regression model to examine the association between the presence of anemia and various risk factors. Individual-level variables included age group, sex, household size category, and malaria rapid diagnostic test (mRDT) results. School-level variables encompassed geographic zone, school altitude, malaria endemicity, and survey year. Each variable was assessed individually in a univariable analysis to estimate crude odds ratios (COR) and 95% Confidence Intervals (CI).

To quantify the contribution of malaria infection to anemia at the population level, we estimated the population attributable risk (PAR) for each survey year. PAR was calculated using malaria RDT positivity as the exposure and anemia as the outcome. We used the standard formula:

*PAR=pe(RR-1)+1pe(RR-1)*

where *pₑ* is the prevalence of malaria infection and *RR* is the risk ratio of anemia comparing malaria-positive to malaria-negative children. Crude risk ratios were derived from observed proportions. Approximate 95% confidence intervals (CIs) for PAR were estimated using the delta method, incorporating uncertainty in both malaria prevalence and the risk ratio.

All variables, including an interaction term between sex and age group, were then included in a multivariable Generalized Linear Mixed Model as described by the regression equation below:

$$logit(\pi_{ijk})\;=\;\beta_{0} + \beta_{1}*AgeGroup_{ijk}\;+\;\beta_{2}*Sex_{ijk}\;+\;\beta_{3}*(AgeGroup_ijk* Sex_ijk )\;+\; \beta_{4}*Residence_{ijk}\;+\;\beta_{5}*Zone_ijk\;+\;\beta_{6}*SurveyYear_ijk+ \beta_{7}*MalariaStrata_{ijk}\;+\;\beta_{8}*HouseholdSize_{ijk}\;+\;\beta_{9}*MalariaInfection_{ijk}\;+\;\mu_{jk}\;+\;v_{k}$$

$\pi_ijk$* represents the probability that individual i in school j in ward k is anemic (1 = Anemic*,* 0 = Not anemic).*

$\beta_0$* is the intercept of the model*,1

$\beta_{1},\beta_{2},...,\beta_{9}\;:$* are the fixed effect coefficients for each predictor variable*.

$\upmu{ik}$* represents the random effect associated with the schools*,* accounting for unobserved heterogeneity between schools*.

*vk:** represents the random effect associated with the ward*,* accounting for unobserved heterogeneity between wards*.

Three levels were incorporated into the logistic regression model: level 1 comprised individual SAC, level 2 represented schools, and level 3 included wards as broader administrative units. The model incorporated random intercepts for both school and ward to account for clustering and unmeasured contextual heterogeneity. This approach allowed the baseline risk of anemia to vary across schools and wards, improving the robustness of fixed-effect estimates. Random slopes were tested but not included in the final model due to convergence challenges and lack of improvement in model fit. This multilevel framework enabled us to account for intra-cluster correlation (ICC) and improved the precision and generalizability of effect estimates across individual- and context-level determinants. Complete case analysis was used to allow comparability between univariable and multivariable regression models.

The Generalized Variance Inflation Factor (GVIF) was used to assess multicollinearity among model covariates, with a threshold of 2 or higher indicating severe multicollinearity. Although GVIF values indicated no significant multicollinearity among covariates (all < 2.5), we also considered conceptual overlap during model specification. Malaria RDT positivity was retained alongside malaria epidemiological strata because they capture distinct levels of exposure individual and contextual, respectively. While mosquito net use was collected in the SMPS instruments, this variable was not included in the multivariable analysis to avoid adjusting for a direct causal factor on the risk of malaria infection. Because stunting (HAZ) and thinness (BAZ) were not measured in 2021, these variables were excluded from the main analysis; however, an additional multivariable logistic regression model was performed on data restricted to surveys from 2019 to 2023 to enable evaluation of the association between anemia and these biometric variables. These decisions were informed by biological plausibility, programmatic relevance, and prior to empirical evidence. Finally, the statistical significance of the interaction term between sex and age group was assessed by comparing the full model with a model without the interaction term using analysis-of-deviance.

All data management and statistical analyses were conducted using R software version 4.4.3. A suite of R packages was utilized to ensure rigorous data cleaning, descriptive analysis, modeling, and visualization. The tidy verse package supported efficient data manipulation and organization, while ggplot2 was used for data visualization. For modeling, the lme4 package was employed to fit multilevel logistic regression models, and sjPlot, broom, and emmeans packages facilitated model output interpretation and presentation. To evaluate multicollinearity and perform diagnostic checks, the car package was applied. Additional epidemiological metrics, such as prevalence and risk estimates, were generated using the epiR package. The use of these tools ensured robust analysis of anemia and associated factors among SAC across the 184 councils in mainland Tanzania.

## Results

### Socio-demographic characteristics

Table 1 presents the socio-demographic characteristics of surveyed SAC from 810 primary schools across mainland Tanzania. A total of 22,104 (46.67%) in 2019, 18,398 (38.84%) in 2021, and 6,864 (14.49%) in 2023 were included for hemoglobin (Hb) concentration estimation, with a median age of 10 years (interquartile range (IQR): 8–12 years). The majority (65.42%) of children were aged 5–11 years, while a smaller proportion (4.21%) fell within the 15–16 age group. The male-to-female distribution remained balanced across all survey years. Based on the school locations, more than half of the children (58.78%) were from rural areas, 56.75% resided in areas with moderate to high malaria transmission risks, and 13.05% had malaria infection. Overall, 81.70% had a normal BMI, 76.29% were not stunted, and 73.48% reported sleeping under a mosquito bed net the night before the survey.

**Table 1:** Social demographic characteristics among surveyed SAC in Tanzania in 2019, 2021, and 2023

Variable	Overall	2019	2021	2023
	*N* = 47,366	*N* = 22,104	*N* = 18,398	*N* = 6,864
**Age group (years)**				
5 to 11	30,986 (65.42%)	13,041 (59.00%)	13,224 (71.88%)	4,721 (68.78%)
12 to 14	14,385 (30.37%)	7,722 (34.93%)	4,724 (25.68%)	1,939 (28.25%)
15 to 16	1,995 (4.21%)	1,341 (6.07%)	450 (2.45%)	204 (2.97%)
**Sex**				
Female	23,692 (50.02%)	11,081 (50.13%)	9,242 (50.23%)	3,369 (49.08%)
Male	23,674 (49.98%)	11,023 (49.87%)	9,156 (49.77%)	3,495 (50.92%)
**Household size**				
<=4	11,736 (25.72%)	4,953 (22.41%)	4,780 (28.38%)	2,003 (29.93%)
5 and above	33,901 (74.28%)	17,150 (77.59%)	12,061 (71.62%)	4,690 (70.07%)
Missing	1729	1	1557	171
**Residence**				
Urban	12,203 (25.76%)	5,991 (27.10%)	4,730 (25.71%)	1,482 (21.59%)
Semi-rural areas	7,320 (15.45%)	3,242 (14.67%)	2,909 (15.81%)	1,169 (17.03%)
Rural	27,843 (58.78%)	12,871 (58.23%)	10,759 (58.48%)	4,213 (61.38%)
**Geographical zones**				
Central	5,112 (10.79%)	2,165 (9.79%)	2,225 (12.09%)	722 (10.52%)
Eastern	8,203 (17.32%)	4,066 (18.39%)	3,101 (16.86%)	1,036 (15.09%)
Lake	12,188 (25.73%)	6,252 (28.28%)	4,329 (23.53%)	1,607 (23.41%)
Northern	6,435 (13.59%)	2,971 (13.44%)	2,560 (13.91%)	904 (13.17%)
Southern	2,294 (4.84%)	866 (3.92%)	1,036 (5.63%)	392 (5.71%)
Southern Highlands	3,691 (7.79%)	1,565 (7.08%)	1,607 (8.73%)	519 (7.56%)
Southwest Highlands	4,345 (9.17%)	1,827 (8.27%)	1,848 (10.04%)	670 (9.76%)
Western	5,098 (10.76%)	2,392 (10.82%)	1,692 (9.20%)	1,014 (14.77%)
**Stunting status**				
Normal	21,885 (76.29%)	17,213 (77.87%)	*	4,672 (70.99%)
Stunted	6,800 (23.71%)	4,891 (22.13%)	*	1,909 (29.01%)
Missing/not collected	18,681	0	18,398	283
**BMI_age**				
Normal	23,404 (81.70%)	18,554 (83.94%)	*	4,850 (74.11%)
Overweight	2,015 (7.03%)	1,096 (4.96%)	*	919 (14.04%)
Thinness	3,229 (11.27%)	2,454 (11.10%)	*	775 (11.84%)
Missing/not collected	18,718	0	18,398	320
**Anemia status**				
Normal	25,243 (53.29%)	11,247 (50.88%)	10,018 (54.45%)	3,978 (57.95%)
Mild	8,948 (18.89%)	4,233 (19.15%)	3,429 (18.64%)	1,286 (18.74%)
Moderate	12,416 (26.21%)	6,094 (27.57%)	4,794 (26.06%)	1,528 (22.26%)
Severe	759 (1.60%)	530 (2.40%)	157 (0.85%)	72 (1.05%)
**Malaria epidemiological. strata**				
Very low	8,600 (18.16%)	3,360 (15.20%)	3,798 (20.64%)	1,442 (21.01%)
Low	11,883 (25.09%)	6,119 (27.68%)	4,430 (24.08%)	1,334 (19.43%)
Moderate	11,270 (23.79%)	4,938 (22.34%)	4,240 (23.05%)	2,092 (30.48%)
High	15,613 (32.96%)	7,687 (34.78%)	5,930 (32.23%)	1,996 (29.08%)
**Bed net use**				
Not slept under-net	11,848 (26.52%)	5,869 (26.55%)	5,270 (31.29%)	709 (12.36%)
Slept under bed net	32,830 (73.48%)	16,234 (73.45%)	11,571 (68.71%)	5,025 (87.64%)
Missing	2688	1	1557	1130
**Malaria infection**				
Negative	41,185 (86.95%)	18,798 (85.04%)	16,312 (88.66%)	6,075 (88.51%)
Positive	6,181 (13.05%)	3,306 (14.96%)	2,086 (11.34%)	789 (11.49%)
**History of fever**				
No fever	36,409 (81.84%)	17,468 (79.10%)	13,192 (83.43%)	5,749 (87.24%)
Yes, had fever	8,078 (18.16%)	4,616 (20.90%)	2,621 (16.57%)	841 (12.76%)
Missing	2879	20	2585	274
**School altitude (metes above sea level)**				
Below 750	13,764 (29.06%)	5,950 (26.92%)	6,000 (32.61%)	1,814 (26.43%)
750 to 1250	18,072 (38.15%)	8,906 (40.29%)	6,617 (35.97%)	2,549 (37.14%)
1250 to 1750	13,508 (28.52%)	6,457 (29.21%)	4,906 (26.67%)	2,145 (31.25%)
Above 1750	2,022 (4.27%)	791 (3.58%)	875 (4.76%)	356 (5.19%)

**Stunting and BMI-for-age data were not collected in 2021

### Hemoglobin concentrations

**Fig. 1:**
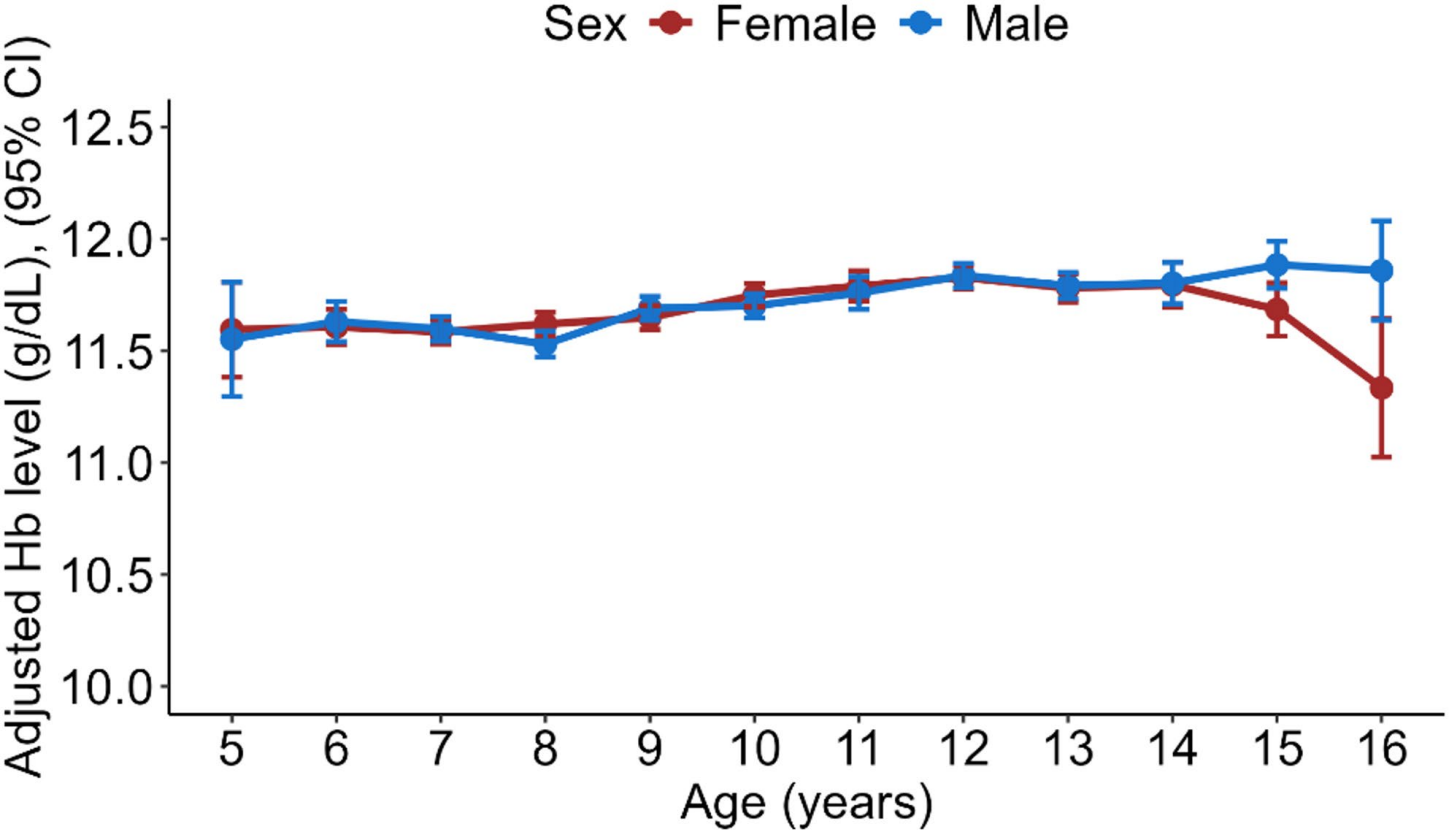
Altitude-adjusted hemoglobin concentration levels among surveyed SAC, stratified by age (5–16 years) and sex (female – red [*n* = 23,692], male – blue [*n* = 23,674]). Error bars indicate 95% confidence intervals in Hb concentration levels among school children aged 5–16 years, unadjusted for school clustering

Figure 1 indicates Hb concentrations among female children generally increased with age, peaking at age 10 (11.75 g/dL, 95%CI: 11.70–11.80) and started to decline to the lowest level at age 16 (11.33 g/dL, 95% CI: 11.03–11.64). Hb concentration levels among male children showed an upward trend with age, reaching the highest mean at age 15 (11.88 g/dL, 95% CI: 11.78–11.99) and the lowest at age 8 (11.53 g/dL, 95% CI: 11.47–11.59). Overall, male children had slightly higher Hb concentrations than female at corresponding ages.

#### Anemia prevalence

**Fig. 2:**
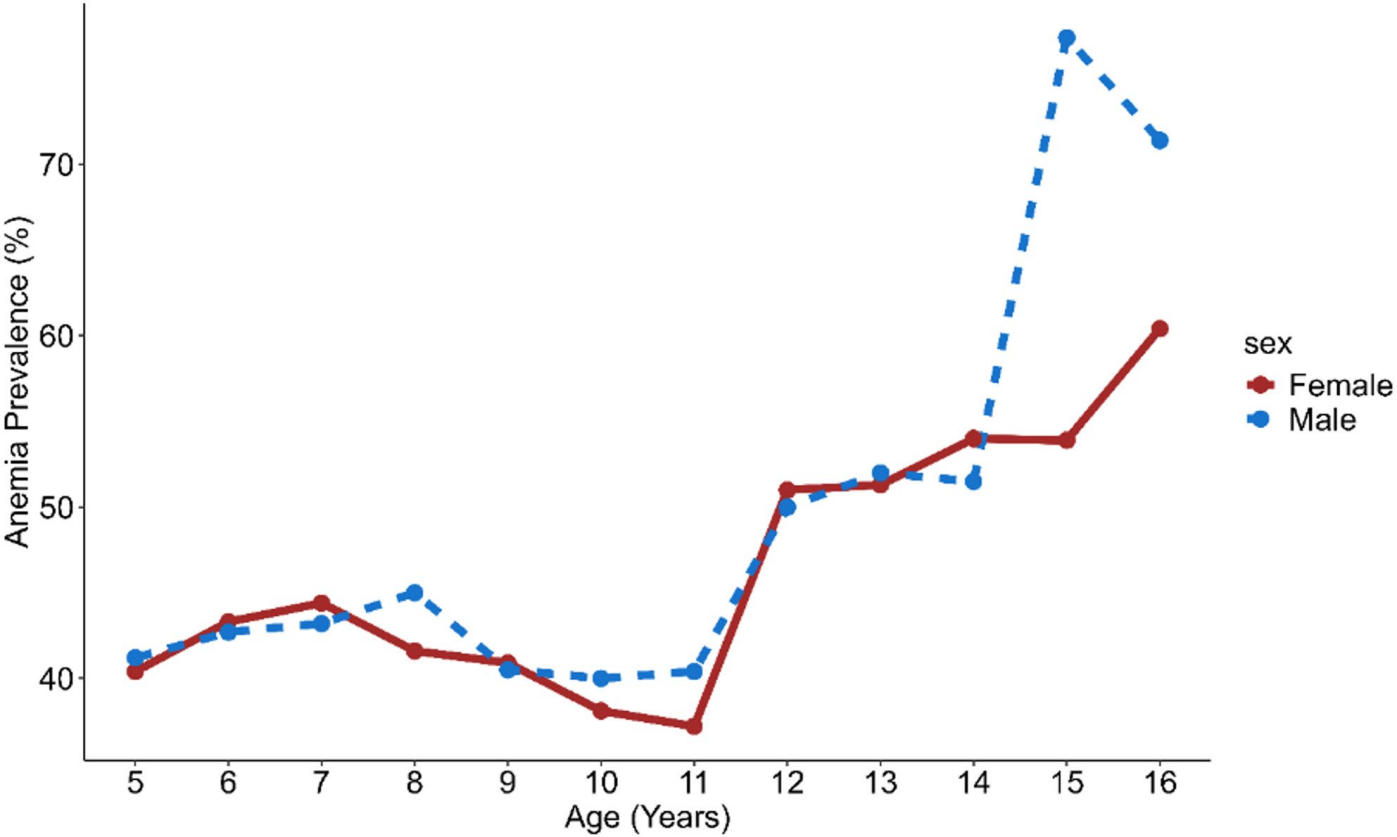
Anemia prevalence among surveyed school-aged children (SAC), across different ages (5–16 years) for both females (red line; *n* = 23,692) and males (blue dashed line; *n* = 23,674) Narrow confidence intervals for younger ages, indicate consistent estimates of anemia prevalence whereas wider confidence intervals suggest greater variability in anemia prevalence, possibly due to individual differences in sample size limitations, existing health conditions, or diet

Overall, the survey found that 45.5% of SAC were anemic, with prevalence rates of 48.04% in 2019, 44.04% in 2021, and 40.94% in 2023 (Table 2). Figure 2 illustrates that anemia prevalence generally increased with age for both males and females. Among females, prevalence started at 40.41% (95% CI: 33.49–47.34) at age 5 and gradually increased to 60.43% (95% CI: 52.30–68.56) by age 16 whereas among males, prevalence began at 41.18% (95% CI: 32.33–50.02) at age 5, peaked at 77.38% (95% CI: 74.73–80.04) at age 15, and slightly declined to 71.37% (95% CI: 65.82–76.92) at age 16. Our findings showed that males aged 15–16 exhibited the highest anemia prevalence, while the lowest prevalence was observed in females aged 10–11.

**Table 2:** Trends of anemia prevalence among SAC in 2019, 2021, and 2023

Variable	Survey years		
	2019	2021	2023
	*N* = 22,104	*N* = 18,398	*N* = 6,864
**Anemia prevalence**	10,619 (48.04%)	8,103 (44.04%)	2,810 (40.94%)
Age group			
5 to 11	5,627 (43.15%)	5,399 (40.83%)	1,777 (37.64%)
12 to 14	4,069 (52.69%)	2,397 (50.74%)	910 (46.93%)
15 to 16	923 (68.83%)	307 (68.22%)	123 (60.29%)
Sex			
Female	5,180 (46.75%)	4,000 (43.28%)	1,336 (39.66%)
Male	5,439 (49.34%)	4,103 (44.81%)	1,474 (42.17%)
Household size category			
<=4	2,222 (44.86%)	2,011 (42.07%)	782 (39.04%)
5 and above	8,397 (48.96%)	5,332 (44.21%)	1,953 (41.64%)
Missing	0	760	75
School altitude group (meters above sea level)			
Below 750	2,643 (44.42%)	2,461 (41.02%)	674 (37.16%)
750 to 1250	4,920 (55.24%)	3,355 (50.90%)	1,170 (46.01%)
1250 to 1750	2,814 (43.58%)	2,019 (41.15%)	857 (39.95%)
Above 1750	242 (30.59%)	268 (29.74%)	109 (30.11%)
Zone			
Central	822 (37.97%)	894 (40.18%)	234 (32.41%)
Eastern	1,600 (39.35%)	1,319 (42.53%)	366 (35.33%)
Lake	3,787 (60.57%)	2,285 (52.78%)	806 (50.16%)
Northern	1,150 (38.71%)	896 (35.00%)	323 (35.73%)
Southern	463 (53.46%)	457 (44.11%)	149 (38.01%)
Southern Highlands	579 (37.00%)	549 (34.16%)	161 (31.02%)
Southwest Highlands	788 (43.13%)	796 (43.07%)	281 (41.94%)
Western	1,430 (59.78%)	907 (53.61%)	490 (48.32%)
ITN use last night			
Not Slept under bed-net	2,763 (47.08%)	2,115 (40.13%)	292 (41.18%)
Sleep Undernet	7,856 (48.39%)	5,228 (45.18%)	2,099 (41.77%)
Missing	0	760	419
Malaria results			
Negative	8,298 (44.14%)	6,753 (41.40%)	2,349 (38.67%)
Positive	2,321 (70.21%)	1,350 (64.72%)	461 (58.43%)
History fevers las two weeks			
No fever	8,182 (46.84%)	5,585 (42.34%)	2,341 (40.72%)
Yes, had fever	2,424 (52.51%)	1,297 (49.48%)	379 (45.07%)
Missing	13	1,221	90
Malaria Epidemiological strata			
Very Low Risk	1,068 (31.79%)	1,189 (31.31%)	439 (30.44%)
Low Risk	2,317 (37.87%)	1,846 (41.67%)	461 (34.56%)
Moderate Risk	2,522 (51.07%)	2,132 (50.28%)	964 (46.08%)
High Risk	4,712 (61.30%)	2,936 (49.51%)	946 (47.39%)
Stunting Status			
Normal	7,996 (46.45%)		1,843 (39.45%)
Stunted	2,623 (53.63%)		828 (43.37%)
Missing			139
BMI age			
Normal	8,891 (47.92%)		1,986 (40.95%)
Overweight	440 (40.15%)		354 (38.52%)
Thinness	1,288 (52.49%)		319 (41.16%)
Missing			151
Residence status			
Urban	2,272 (37.92%)	1,951 (41.25%)	519 (35.02%)
Semi-rural	1,419 (43.77%)	1,161 (39.91%)	496 (42.43%)
Rural	6,928 (53.83%)	4,991 (46.39%)	1,795 (42.61%)

Anemia prevalence is relatively high across all age groups, with values exceeding 40% in most cases. Older adolescents (15–16 years) consistently had the highest anemia rates, while younger children (5–11 years) had the lowest rates. Males showed slightly higher prevalence than females. Children in larger households and rural areas had increased anemia risk (Table 2).

The population attributable risk (PAR) of anemia due to malaria infection declined over time. In 2019, PAR was estimated at 8.1% (95% CI: 7.2–9.0), decreasing to 6.0% (95% CI: 5.2–6.9) in 2021 and 5.5% (95% CI: 4.3–6.8) in 2023.

### Region, council, and school-level anemia trends by severity

**Fig. 3:**
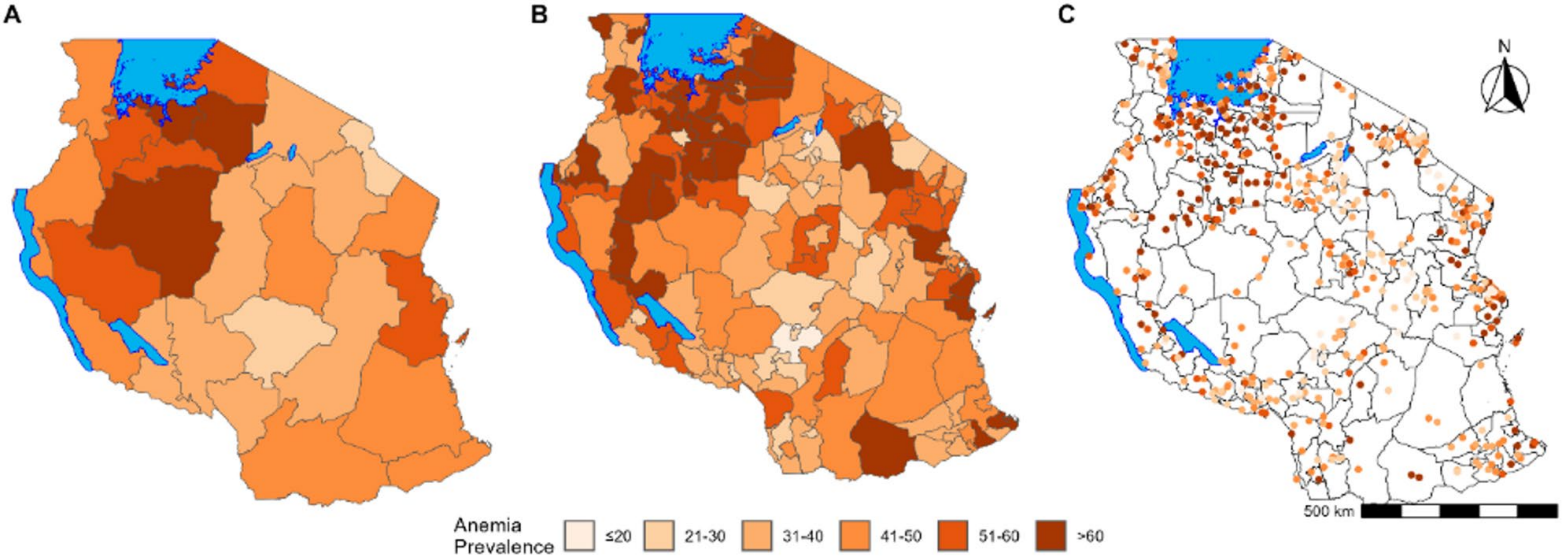
Maps of mainland Tanzania indicating severity of anemia by region (**A**), council (**B**), and schools (**C**) as shown by color pallets indicating mild to severe anemia. The base map was generated using a shapefile for Tanzania that included regional and council boundaries [33]. Geographic coordinates (latitude and longitude) of the surveyed schools were captured during data collection. Data used to generate these maps were plotted and visualized using R software version 4.4.3

Figure 3 illustrates the prevalence of anemia at the regional (A), council (B), and school (C) levels. The results reveal a geographical divide, with higher anemia prevalence in the Western, Eastern, and Southern regions, while the central corridor exhibits lower prevalence. However, when analyzed at the council level, significant heterogeneity in anemia prevalence was observed within regions. This variation became even more pronounced when examined at the school level, highlighting localized disparities in anemia burden.

**Fig. 4:**
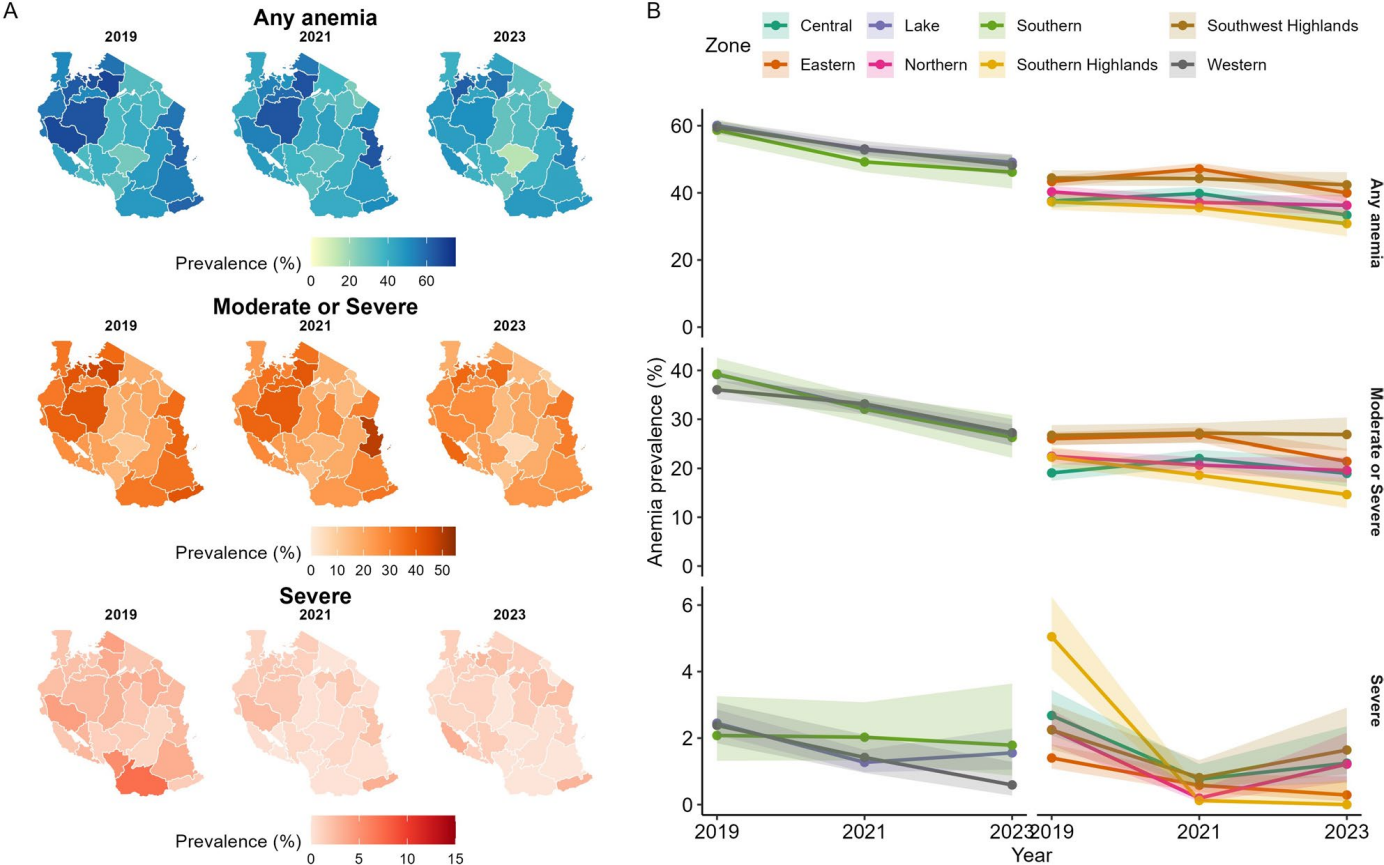
Trends and spatial patterns in anemia prevalence among SAC in Tanzania, 2019, 2021, and 2023. Figure 4 (**A**); maps showing region-level prevalence of any anemia, moderate or severe anemia, and severe anemia across survey years. The base map was generated using a Tanzania shapefile containing regional and council boundaries [33]. Anemia classification was based on altitude-adjusted Hb thresholds defined by 2024 WHO criteria. Figure 4 (**B**) Time-series plots of anemia prevalence by zone, stratified by severity. Shaded ribbons represent 95% binomial confidence intervals

Anemia prevalence also declined significantly between 2019 and 2023 across all severity categories (Fig. 4). The largest absolute reductions were observed in milder forms due to their higher baseline prevalence, while severe anemia also declined substantially in relative terms. Notably, zones with the highest malaria burdens nationally including the Lake, Western, and Southern zones had among the highest anemia prevalence in 2019 and showed the most consistent downward trends, particularly for moderate and severe forms.

### Generalized multilevel mixed-effect model analysis of anemia among SAC

Table 3 presents the results of the multivariable logistic regression analysis assessing risk factors associated with anemia among SAC in mainland Tanzania, including odds ratios (OR), 95% confidence intervals (CI), and p-values from both univariable and multivariable models. The variance attributable to differences between schools was 0.30, indicating clustering of anemia outcomes within schools, while 0.24 variance was due to differences between wards, reflecting additional clustering at the ward level. The intraclass correlation coefficient (ICC) of 0.14 indicates that 14% of the total variance in anemia is explained by clustering at school and ward levels, supporting the use of a multilevel model. The analysis included 45,637 pupils from 764 schools and 761 wards, representing a broad sample. Fixed effects explained 7.4% of the variance in anemia, increasing to 20.6% when including both fixed and random effects.

**Table 3:** Univariable and multivariable mixed effects logistic regression analysis of factors associated with anemia among school-age children in mainland Tanzania in 2019, 2021, and 2023, *N* = 45,637

Variables	Univariable			Multivariable	
	COR^1^ (95% CI)^2^	*P*-value		AOR^3^ (95% CI)^2^	*P*-value
Sex			Sex in 5–11 years group		
Female	1	-	Female	1	-
Male	1.10 (1.06–1.14)	**< 0.001**	Male	1.04 (0.97–1.12)	0.660
			**Sex in 12–14 years group**		
			Female	1	-
			Male	0.96 (0.87–1.07)	0.914
			**Sex in 15–16 years group**		
			Female	1	-
			Male	2.96 (2.18–4.02)	**< 0.001**
**Age group (years)**			**Age group (years) in Females**		
5–11	1	-	5–11	1	-
12–14	1.46 (1.40–1.53)	**< 0.001**	12–14	1.49 (1.36–1.63)	**< 0.001**
15–16	2.67 (2.40–2.97)	**< 0.001**	15–16	1.39 (1.10–1.76)	**< 0.001**
			**Age group (years) in Males**		
			5–11	1	**-**
			12–14	1.38 (1.26–1.51)	**< 0.001**
			15–16	3.96 (3.19–4.93)	**< 0.001**
**Household size**			**Household size**		
≤ 4 members	1	-	≤ 4 members	1	-
≥ 5 more members	1.02 (0.97–1.07)	0.496	≥ 5 more members	0.97 (0.92–1.01)	0.160
**Geographical zone**			**Geographical zone**		
Central	1	-	Central	1	-
Eastern	1.53 (1.21–1.94)	**< 0.001**	Eastern	1.56 (1.22–1.99)	**< 0.001**
Lake	2.39 (1.92–2.99)	**< 0.001**	Lake	1.77 (1.38–2.26)	**< 0.001**
Northern	1.08 (0.85–1.38)	0.527	Northern	1.13 (0.89–1.43)	0.310
Southern	2.03 (1.49–2.78)	**< 0.001**	Southern	1.65 (1.18–2.30)	**0.003**
Southern Highlands	0.96 (0.72–1.28)	0.795	Southern Highlands	0.89 (0.67–1.17)	0.398
Southwest Highland	1.44 (1.10–1.89)	**0.007**	Southwest Highland	1.23 (0.95–1.59)	0.122
Western	2.42 (1.86–3.16)	**< 0.001**	Western	1.78 (1.34–2.37)	**< 0.001**
**Malaria RDT result**			**Malaria RDT result**		
Negative	1	-	Negative	1	-
Positive	2.01 (1.88–2.16)	**< 0.001**	Positive	1.88 (1.75–2.01)	**< 0.001**
**Malaria Epidemiological strata**			**Malaria Epidemiological strata**		
Very low	1	-	Very low	1	-
Low	1.42 (1.25–1.61)	**< 0.001**	Low	1.21 (1.05–1.40)	**0.007**
Moderate	2.12 (1.84–2.44)	**< 0.001**	Moderate	1.49 (1.26–1.76)	**< 0.001**
High	2.24 (1.94–2.459)	**< 0.001**	High	1.24 (1.02–1.50)	**0.027**
**Residence status**			**Residence status**		
Urban areas	1	-	Urban areas	1	-
Semi-rural areas	1.05 (0.86–1.30)	0.618	Semi-rural areas	1.08 (0.88–1.31)	0.473
Rural area	1.45 (1.24–1.69)	**< 0.001**	Rural area	1.37 (1.17–1.60)	**< 0.001**
**Survey year**			**Survey year**		
2019	1	-	2019	1	-
2021	0.83 (0.79–0.87)	**< 0.001**	2021	0.91 (0.87–0.96)	**< 0.001**
2023	0.69 (0.65–0.74)	**< 0.001**	2023	0.75 (0.70–0.80)	**< 0.001**

^1^Crude odds ratio

^2^95% Confidence Interval

^3^Adjusted odds ratio; adjusted using age, sex, age-sex interaction, household size, geographical zone, malaria RDT result, malaria epidemiological strata, residence status, and survey year. Random effects: σ^2^ = 3.29, τ_00school_registration_number_ = 0.30, τ_00ward_ = 0.24, ICC = 0.14, N _school_registration_number_ = 764, N _ward_ = 761, Marginal R^2^ = 0.074, Conditional R^2^ = 0.206

A statistically significant interaction between sex and age group was observed (*p* < 0.001), indicating that the association between sex and anemia varied by age. Among younger children, males and females had similar odds of anemia (SAC 5–11 years: Adjusted Odds Ratio [AOR] = 1.04 [95%CI: 0.97–1.12, *p* = 0.660]; SAC 12–14 years: AOR = 0.96 [95%CI: 0.87–1.07, *p* = 0.914]). However, among older children (age 15–16 years), males had almost three times the odds of anemia compared to femals (AOR = 2.96 [95%CI: 2.18–4.02, *p* < 0.001]). In addition to age and sex, statistically significant variables within the multivariable logistic regression model included geographical zone, malaria RDT result, malaria epidemiological strata, residence status, and survey year. SAC in Eastern (AOR = 1.56 [95%CI: 1.22–1.99]), Lake (AOR = 1.77 [95%CI: 1.38–2.26], Southern (AOR = 1.65 [95%CI: 1.18–2.30]), and Western (AOR = 1.78 [95%CI: 1.34–2.37]) zones all had statistically significant higher odds of anemia than SAC in the Central zone. SAC living in rural areas had 37% higher odds of anemia compared to SAC in urban areas (AOR = 1.37 [95%CI: 1.17–1.60]). Malaria risk and current infection were also significant predictors of anemia. A positive malaria RDT result was associated with 88% higher odds of anemia compared to a negative result (AOR = 1.88 [95%CI: 1.75–2.01]). SAC living in areas with higher malaria epidemiologic risk had a greater odd of anemia compared to SAC in very low risk strata although this difference was greatest in moderate strata (AOR = 1.49 [95%CI: 1.26–1.76]). The multivariable analysis also supported evidence of a decreasing trend in anemia prevalence over time (2021 vs. 2019: AOR = 0.91 [95%CI: 0.87–0.96]; 2023 vs. 2019: AOR = 0.75 [95%CI: 0.70–0.80]). Household size showed no evidence of association with anemia.

An additional analysis was performed to assess the association of anemia with biometric indicators (BMI for Age and stunting) which were measured in 2019 and 2023, but not 2021 (S1: Table 1). When excluding the 2021 survey, stunting was associated with anemia: SAC with stunted growth had slightly higher odds of anemia compared to non-stunted growth (AOR = 1.13 [95%CI: 1.06–1.21]), even after adjusting for other factors. details advanced analysis performed without 2021 survey data that did not report BMI for Age and stunting. Although a statistically significant association between anemia and BMI was observed on univariable analysis, this association disappeared after multivariable adjustment.

## Discussion

This study analyzed data from three nationwide surveys conducted in 2019, 2021, and 2023, which reveal persistently high but progressively declining anemia prevalence among SAC in mainland Tanzania. This downward trajectory aligns with findings from a multilevel trend analysis spanning 2015 to 2023, which reported a significant reduction in malaria prevalence among SAC [27]. The observed heterogeneity and similar patterns of both anemia and malaria further suggest that the observed decline in anemia is, in part, attributable to reduced malaria burden in these populations [27]. These findings reinforce the critical role of malaria control interventions in alleviating anemia among SAC. Moreover, our multivariable logistic regression analyses identified several independent predictors of anemia, including age, sex, malaria infection, malaria epidemiological risk strata, rural residence, stunting, and geographic zone, underscoring the multifactorial nature of anemia risk among SAC.

Anemia remains a major public health concern, particularly among children and adolescents, due to its negative impacts on cognitive function, academic performance, and overall well-being [2]. Our study found that children with malaria infection were nearly twice as likely to have anemia compared to non-infected children, consistent with previous reports in the country in under-five children [34]. SAC from schools in moderate-to-high malaria transmission areas had a significantly higher risk of anemia, aligning with previous studies [7, 12]. The patterns of anemia prevalence closely mirrored malaria prevalence trends in Tanzania, with higher rates in the Lake, Western, and Southern zones [20]. Malaria parasites have been reported to increase the host’s metabolic demand for nutrients, further worsening anemia [35].

Malaria remains a major contributor to anemia in Tanzania, particularly in high transmission areas. Our findings align with previous research demonstrating that children living in regions with higher malaria endemicity exhibit increased anemia prevalence due to hemolysis and impaired erythropoiesis caused by Plasmodium falciparum infection [36, 37]. For example, Mwaiswelo et al. (2021) reported that children in councils with higher malaria prevalence had significantly greater odds of anemia, emphasizing the role of malaria control in reducing anemia burden [36]. Similarly, asymptomatic malaria infections have been associated with lower hemoglobin levels, contributing to anemia even in the absence of clinical symptoms [38]. These findings underscore the importance of integrating malaria control strategies, such as insecticide-treated nets and seasonal chemoprevention, into anemia reduction programs.

Temporal variations in anemia prevalence likely reflect changes in malaria control efforts, nutrition, and broader socioeconomic conditions over time. Mainland Tanzania scaled up multiple malaria interventions between 1999 and 2010, leading to marked declines in severe anemia and under-five mortality [37]. Our analysis, which incorporates data from multiple survey rounds between 2019 and 2023, suggests a continued reduction in anemia prevalence corresponding with intensified malaria control and broader public health improvements. This trend is consistent with earlier analyses showing declines in malaria prevalence among SAC following the expansion of intervention coverage [27]. Notably, declines in anemia were observed across most regions and all severity levels, with the most sustained reductions occurring in zones with the highest malaria burden: Lake, Western, and Southern. These areas had among the highest baseline anemia prevalence and showed consistent declines, reinforcing the notion that malaria dynamics remain a key driver of anemia risk in SAC and suggesting that sustained malaria control may be yielding broader health benefits. However, persistent pockets of high anemia burden remain, underscoring the importance of targeted, equity-focused strategies. Future work should further explore the etiology of anemia by severity and assess the differential impact of overlapping interventions in diverse transmission and nutritional settings.

Studies from malaria-endemic areas underscore the multifactorial drivers of anemia among SAC. A study conducted in Kilosa district council, Morogoro, Tanzania showed that anemia was higher in children not using mosquito nets and among those who had malaria infection [39]. In Kenya, Brooker et al. [40] and Halliday et al. [41] demonstrated that malaria infection was a significant risk factor for anemia in among school children, and that school-based malaria interventions can improve hemoglobin levels and reduce anemia prevalence; highlighting the importance of integrating malaria control with nutritional and educational interventions to address anemia more effectively. Similarly, a multi-country analysis including Ethiopia, highlighted how regional malaria epidemiology and dietary diversity interact to shape anemia risk, with higher burdens observed in areas of prolonged malaria transmission and limited access to iron-rich foods [17]; emphasizing the synergistic role of insecticide-treated nets and school-based iron supplementation. These findings reinforce the importance of multisectoral approaches to address anemia in SAC populations across sub-Saharan Africa.

Anemia prevalence was significantly higher among older SAC (12–14 and 15–16 years) compared to younger children (5–11 years). This may be attributed to physiological changes during puberty, which increase the demand for iron and other essential nutrients [42–44]. Male SAC had a higher prevalence of anemia than females within the same age groups, contrasting with some studies that found higher risks among females [6, 45]. In females, iron requirements rise as they approach menarche, while in males, the onset of testosterone production stimulates erythropoiesis, which may influence anemia risk [6]. Insufficient iron intake during rapid growth phases may contribute to these observed differences.

PAR estimates clarify the divergence between anemia and malaria trends. Although malaria remained significantly associated with anemia, its population-level contribution was modest and declined over time (≈ 5–8%), indicating that over 90% of anemia cases were attributable to non-malarial causes. The larger reduction in anemia compared with malaria infection likely reflects combined effects of improved nutrition, deworming, school health interventions, and broader socioeconomic changes. These findings underscore the need for integrated child health strategies, as malaria control alone is insufficient to address anemia among school-aged children [46, 47].

Nutritional deficiencies, parasitic infections, and sanitation also contribute significantly to anemia burden [36]. In our analysis of surveys in 2019 and 2023, SAC with stunted growth had higher odds of anemia compared to their non-stunted counterparts, suggesting long-term nutritional deficiencies might influence risk of anemia, consistent with previous studies [48, 49]. Anemia and stunting pose significant challenges to child survival and strain the healthcare system. The association between stunting and anemia is well-documented, with both conditions sharing common environmental, socio-economic, and dietary risk factors [14, 50–52]. Diets low in bioavailable iron and inadequate dietary iron intake often accompany other micronutrient deficiencies, exacerbating anemia risk [11, 53, 54]. Additionally, malnutrition can hinder nutrient absorption and utilization, further exacerbating the risk of iron deficiency and anemia.

This study confirms a strong association between rural residence and higher odds of anemia among SAC. In both univariable and multivariable analyses, children in rural areas had higher odds of anemia than those in urban areas, with a crude odds ratio of 1.45 (95% CI: 1.24–1.69) and an adjusted odds ratio of 1.37 (95% CI: 1.17–1.60). This association remained statistically significant after adjustment for confounding variables, indicating persistent rural–urban disparities in anemia burden [54–56]. In contrast, children in semi-rural areas did not show significantly different odds of anemia compared to those in urban areas. These findings highlight substantial geographic disparities in anemia burden, with rural children facing disproportionate risk [55–57]. Contributing factors include limited access to diverse, iron-rich diets, lower household income, and poor market connectivity [55, 56, 58]. Rural settings also tend to have higher prevalence of infectious diseases such as malaria, schistosomiasis, and soil-transmitted helminths conditions strongly associated with anemia among SAC [54, 58].

Access to preventive and curative health services in rural areas is often constrained by systemic barriers, including fewer health facilities, poor transportation networks, stock-outs of essential supplies, and workforce shortages. These limitations reduce uptake of services such as deworming, iron supplementation, and timely malaria treatment, exacerbating the risk of anemia [54–56]. Additionally, disparities in water, sanitation, hygiene, food security, and parental education further contribute to poorer health outcomes in rural populations [54, 58]. Consistent with findings from Tanzania and across sub-Saharan Africa, this analysis reinforces that rural SAC are particularly vulnerable to anemia, primarily due to limited access to essential health and nutrition services [54, 56, 59].

### Strengths and Limitations

Our study represents the largest nationally representative assessment of anemia among SAC in Tanzania, using data from 674 schools across 184 councils and 26 regions. The multilevel modeling approach accounted for clustering at the school and ward levels, strengthening the reliability of the estimates. Integration of biological (Hb concentration, malaria RDTs), demographic, and spatial data allowed for a comprehensive risk assessment.

Nevertheless, several limitations must be acknowledged. First, the school-based sampling excluded out-of-school children, potentially underestimating anemia prevalence, particularly among vulnerable populations with lower school attendance [60]. Second, anthropometric indicators such as BMI and stunting were excluded from the final model due to missing data across survey rounds, limiting our assessment of nutritional contributions to anemia. Nutritional deficiencies, including iron, folate, and vitamin A, are well-established anemia determinants [61]. While their exclusion may have introduced residual confounding, additional analyses excluding the 2021 survey suggest stunting and BMI do not act as major confounders of the other factors in the regression model.

Hb concentration was measured using the HemoCue Hb 201+, a validated tool for field surveys. However, this device may underestimate Hb values by ~ 0.53 g/dL compared to laboratory methods, with a standard deviation of ± 1.01 g/dL [62], potentially misclassifying borderline anemia cases. Additionally, survey data were collected from July to October, typically during the dry season when malaria transmission is lower in many regions. This seasonal bias may have led to underestimation of malaria-associated anemia, especially in areas with unstable transmission [63].

We also recognize potential conceptual overlap between malaria RDT results and epidemiological strata. However, these variables were retained due to their unique contribution to understanding malaria exposure at both individual and contextual levels, respectively.

Although there is a time gap between survey rounds and some children may have been sampled more than once, the anonymization of pupils prevents linkage of individuals across rounds, limiting longitudinal analysis at the individual level. In addition, detailed dietary intake and micronutrient data were not consistently available across survey rounds, limiting assessment of the contribution of nutritional deficiencies to anemia trends. Information on pubertal status was also not collected, which may influence hemoglobin levels and partially explain observed age- and sex-specific differences in anemia.

Despite these limitations, the study’s strengths include its national scope, integration with routine surveillance, and multilevel design to provide a robust source of evidence for guiding targeted interventions. Findings highlight the need for anemia control strategies tailored to geographic, nutritional, and malaria transmission contexts across Tanzania.

## Conclusion

The Tanzania School Malaria Parasitological Survey (SMPS), conducted in 2019, 2021, and 2023, is the only nationwide survey that integrates nutrition indices with the most up-to-date data on hemoglobin concentration levels and associated risk factors among SAC aged 5–16 years. Anemia remains a significant public health challenge in this population, with notable geographical variation as well as sex- and age-specific differences. Our findings highlight the urgent need for integrated interventions targeting malaria control, nutritional improvement, and helminth prevention, with a focus on high-risk groups and localized settings.

We call on stakeholders, policymakers, and health professionals to leverage these findings by strengthening routine anemia screening in schools; tailoring interventions to address specific risk factors across regions, councils, and schools, particularly for older male children; integrating anemia control efforts with existing malaria and nutrition programs; and enhancing surveillance systems to include comprehensive data on nutritional status and socioeconomic factors.

Future research should focus on investigating the underlying causes of geographical variation in anemia prevalence; evaluating the effectiveness of different intervention strategies in reducing anemia in SAC; assess the long-term impact of anemia on educational outcomes and economic productivity; and conducting cost-effectiveness analyses of anemia interventions in this population.

## Supplementary Information

Supplementary Material 1.

## Data Availability

The data used for these outputs was sourced from the national school-based surveillance system, established in 2014-15. This is primarily programmatic surveillance data rather than research-specific data. Most of the data utilized in this analysis is presented in the corresponding figures, tables, and supplementary materials. Comprehensive reports from previous TSMPS surveys are available at nmcp.go.tz/index.php/publication.However, due to current policies and restrictions on sharing government data, requests for additional data supporting this study’s findings can be directed to the Tanzania Ministry of Health via https://www.moh.go.tz/contact or by emailing the Permanent Secretary at ps@afya.go.tz.
