# Supplementary material for: Trends in prevalence and predictors of anemia among school-aged children in Tanzania: analysis of 2019–2023 cross-sectional surveys using a multilevel logistic regression model
Source: BMC Public Health. 2026 May 27;26:2270. doi: 10.1186/s12889-026-27799-y (PMC13440171; doi:10.1186/s12889-026-27799-y)
Supplement: Supplementary file 1 [file 12889_2026_27799_MOESM1_ESM.docx]

**Supplementary Information (SI)**

S1: Table 1

This table presents both univariable and multivariable logistic regression analyses of anemia among SAC in Tanzania without 2021 TSMPS, showing Crude Odds Ratios (COR) and Adjusted Odds Ratios (AOR) with corresponding 95% Confidence Intervals (CI) and P-values. The table shows anemia prevalence as the outcome variable, with associated risk factors assessed for their association with the outcome. N = 28,382

| **Variables** | **Univariable** | | **Multivariable – Main Model** | | | **Multivariable – Adjusting for Stunting and BMI** | | |
| --- | --- | --- | --- | --- | --- | --- | --- | --- |
|  | **COR^1^ (95% CI)^2^** | **P-value** |  | **AOR^3^ (95% CI)^2^** | **P-value** |  | **AOR^4^ (95% CI)^2^** | **P-value** |
| **Sex** |  |  | **Sex in 5-11 years group** |  |  | **Sex in 5-11 years group** |  |  |
| Female | 1 | - | Female | 1 | - | Female | 1 | - |
| Male | 1.12 (1.06 – 1.17) | **<0.001** | Male | 1.05 (0.95 – 1.16) | 0.710 | Male | 1.05 (0.95 – 1.16) | 0.689 |
|  |  |  | **Sex in 12-14 years group** |  |  | **Sex in 12-14 years group** |  |  |
|  |  |  | Female | 1 | - | Female | 1 | - |
|  |  |  | Male | 0.97 (0.85 – 1.11) | 0.987 | Male | 0.96 (0.84 – 1.09) | 0.928 |
|  |  |  | **Sex in 15-16 years group** |  |  | **Sex in 15-16 years group** |  |  |
|  |  |  | Female | 1 | - | Female | 1 | - |
|  |  |  | Male | 2.77 (1.94 – 3.96) | **<0.001** | Male | 2.64 (1.85 – 3.79) | **<0.001** |
| **Age group (years)** |  |  | **Age group (years) in Females** |  |  | **Age group (years) in Females** |  |  |
| 5–11 | 1 | - | 5–11 | 1 | 1 | 5–11 | 1 | 1 |
| 12–14 | 1.46 (1.38 – 1.55) | **<0.001** | 12–14 | 1.48 (1.32 – 1.67) | **<0.001** | 12–14 | 1.46 (1.30 – 1.64) | **<0.001** |
| 15–16 | 2.72 (2.40 – 3.09) | **<0.001** | 15–16 | 1.46 (1.11 – 1.93) | **0.001** | 15–16 | 1.45 (1.10 – 1.91) | **0.002** |
|  |  |  | **Age group (years) in Males** |  |  | **Age group (years) in Males** |  |  |
|  |  |  | 5–11 | 1 | **-** | 5–11 | 1 | **-** |
|  |  |  | 12–14 | 1.37 (1.22 – 1.54) | **<0.001** | 12–14 | 1.33 (1.18 – 1.49) | **<0.001** |
|  |  |  | 15–16 | 3.85 (2.99 – 4.96) | **<0.001** | 15–16 | 3.64 (2.82 – 4.70) | **<0.001** |
| **Household size** |  |  | **Household size** |  |  | **Household size** |  |  |
| ≤ 4 members | 1 | - | ≤ 4 members | 1 | - | ≤ 4 members | 1 | - |
| ≥ 5 more members | 1.01 (0.95 – 1.08) | 0.734 | ≥ 5 more members | 0.95 (0.89 – 1.01) | 0.112 | ≥ 5 more members | 0.95 (0.89 – 1.01) | 0.103 |
| **Geographical zone** |  |  | **Geographical zone** |  |  | **Geographical zone** |  |  |
| Central | 1 | - | Central | 1 | - | Central | 1 | - |
| Eastern | 1.70 (1.27 – 2.27) | **<0.001** | Eastern | 1.82 (1.33 – 2.48) | **<0.001** | Eastern | 1.84 (1.35 – 2.51) | **<0.001** |
| Lake | 3.06 (2.32 – 4.02) | **<0.001** | Lake | 2.22 (1.63 – 3.03) | **<0.001** | Lake | 2.26 (1.66 – 3.08) | **<0.001** |
| Northern | 1.31 (0.97 – 1.77) | 0.083 | Northern | 1.37 (1.03 – 1.84) | **0.033** | Northern | 1.39 (1.04 – 1.86) | **0.028** |
| Southern | 2.46 (1.67 – 3.62) | **<0.001** | Southern | 2.03 (1.34 – 3.09) | **0.001** | Southern | 2.05 (1.35 – 3.12) | **0.001** |
| Southern Highlands | 1.03 (0.73 – 1.46) | 0.866 | Southern Highlands | 0.93 (0.66 – 1.31) | 0.68 | Southern Highlands | 0.93 (0.66 – 1.31) | 0.685 |
| Southwest Highland | 1.58 (1.14 – 2.20 | **0.006** | Southwest Highland | 1.34 (0.97 – 1.85) | 0.073 | Southwest Highland | 1.35 (0.98 – 1.87) | 0.066 |
| Western | 2.87 (2.08 – 3.96) | **<0.001** | Western | 2.10 (1.47 – 3.00) | **<0.001** | Western | 2.13 (1.49 – 3.05) | **<0.001** |
| **Malaria RDT result** |  |  | **Malaria RDT result** |  |  | **Malaria RDT result** |  |  |
| Negative | 1 | - | Negative | 1 | - | Negative | 1 | - |
| Positive | 2.02 (1.85 – 2.20) | **<0.001** | Positive | 1.86 (1.70 – 2.03) | **<0.001** | Positive | 1.85 (1.69 – 2.02) | **<0.001** |
| **Malaria Epidemiological strata** |  |  | **Malaria Epidemiological strata** |  |  | **Malaria Epidemiological strata** |  |  |
| Very low | 1 | - | Very low | 1 | - | Very low | 1 | - |
| Low | 1.41 (1.19 – 1.68) | **<0.001** | Low | 1.21 (1.00 – 1.46) | 0.052 | Low | 1.21 (0.99 – 1.46) | 0.056 |
| Moderate | 2.31 (1.92 – 2.78) | **<0.001** | Moderate | 1.55 (1.24 – 1.93) | **<0.001** | Moderate | 1.54 (1.23 – 1.92) | **<0.001** |
| High | 2.68 (2.23 – 3.23) | **<0.001** | High | 1.26 (0.98 – 1.62) | 0.07 | High | 1.25 (0.97 – 1.61) | 0.085 |
| **Residence status** |  |  | **Residence status** |  |  | **Residence status** |  |  |
| Urban areas | 1 | - | Urban areas | 1 | - | Urban areas | 1 | - |
| Semi-rural areas | 1.07 (0.83 – 1.37) | 0.622 | Semi-rural areas | 1.10 (0.86 – 1.41) | 0.433 | Semi-rural areas | 1.10 (0.86 – 1.41) | 0.466 |
| Rural area | 1.58 (1.31 – 1.91) | **<0.001** | Rural area | 1.50 (1.23 – 1.83) | **<0.001** | Rural area | 1.48 (1.22 – 1.81) | **<0.001** |
| **Survey year** |  |  | **Survey year** |  |  | **Survey year** |  |  |
| 2019 | 1 | - | 2019 | 1 | - | 2019 | 1 | - |
| 2023 | 0.67 (0.63 – 0.72) | **<0.001** | 2023 | 0.76 (0.71 – 0.81) | **<0.001** | 2023 | 0.73 (0.68 – 0.78) | **<0.001** |
| **Stunting Status** |  |  |  |  |  | **Stunting Status** |  |  |
| Non-stunted | 1 | - |  |  |  | Non-stunted | 1 | - |
| Stunted | 1.27 (1.19 – 1.35) | **<0.001** |  |  |  | Stunted | 1.13 (1.06 – 1.21) | **<0.001** |
| **BMI^5^** |  |  |  |  |  | **BMI^5^** |  |  |
| Underweight | 1.15 (1.06 – 1.25) | **0.001** |  |  |  | Underweight | 1.07 (0.98 – 1.17) | 0.122 |
| Normal | 1 | - |  |  |  | Normal | 1 | - |
| Overweight | 0.84 (0.75 – 0.93) | **0.001** |  |  |  | Overweight | 0.94 (0.85 – 1.06) | 0.312 |

*^1^Crude odds ratio*
*^2^95% Confidence Interval*
*^3^Adjusted odds ratio; adjusted using age, sex, age-sex interaction, household size, geographical zone, malaria RDT result, malaria epidemiological strata, residence status, and survey year. Random effects:* σ^2^ = 3.29, τ_00_ _school_registration_number_ = 0.42, τ_00_ _ward_ = 0.35, ICC = 0.19, N _school_registration_number_ = 740, N _ward_ = 740, Marginal R^2^ = 0.088, Conditional R^2^ = 0.261
*^4^Adjusted odds ratio; adjusted using age, sex, age-sex interaction, household size, geographical zone, malaria RDT result, malaria epidemiological strata, residence status, survey year, stunting status, and body mass index . Random effects:* σ^2^ = 3.29, τ_00_ _school_registration_number_ = 0.42, τ_00_ _ward_ = 0.35, ICC = 0.19, N _school_registration_number_ = 740, N _ward_ = 740, Marginal R^2^ = 0.088, Conditional R^2^ = 0.262
*^5^*Body mass index (kg/m^2^), categories adjusted for age and sex
